# Widespread Cotranslational Formation of Protein Complexes

**DOI:** 10.1371/journal.pgen.1002398

**Published:** 2011-12-01

**Authors:** Caia D. S. Duncan, Juan Mata

**Affiliations:** Department of Biochemistry, University of Cambridge, Cambridge, United Kingdom; Sanford-Burnham Medical Research Institute, United States of America

## Abstract

Most cellular processes are conducted by multi-protein complexes. However, little is known about how these complexes are assembled. In particular, it is not known if they are formed while one or more members of the complexes are being translated (cotranslational assembly). We took a genomic approach to address this question, by systematically identifying mRNAs associated with specific proteins. In a sample of 31 proteins from *Schizosaccharomyces pombe* that did not contain RNA–binding domains, we found that ∼38% copurify with mRNAs that encode interacting proteins. For example, the cyclin-dependent kinase Cdc2p associates with the *rum1* and *cdc18* mRNAs, which encode, respectively, an inhibitor of Cdc2p kinase activity and an essential regulator of DNA replication. Both proteins interact with Cdc2p and are key cell cycle regulators. We obtained analogous results with proteins with different structures and cellular functions (kinesins, protein kinases, transcription factors, proteasome components, etc.). We showed that copurification of a bait protein and of specific mRNAs was dependent on the presence of the proteins encoded by the interacting mRNAs and on polysomal integrity. These results indicate that these observed associations reflect the cotranslational interaction between the bait and the nascent proteins encoded by the interacting mRNAs. Therefore, we show that the cotranslational formation of protein–protein interactions is a widespread phenomenon.

## Author Summary

Most proteins do not function in isolation. Instead, they associate with other proteins to form complexes. Little is known about the assembly of protein complexes within cells. One possibility is that proteins are completely synthesised before they bind to each other. An alternative is that proteins attach to each other as they are being translated in the ribosome (called cotranslational assembly). To investigate if cells use cotranslational assembly to form complexes, we identified mRNAs associated with specific proteins. The expectation is that if protein A binds to protein B as protein B is being translated, A will associate indirectly to the mRNA encoding B. Indeed, we found that for ∼40% of proteins (out of a sample of over 30) this was the case. Proteins associated with a small number of mRNAs, most of which encoded known or predicted interacting proteins. We found examples of this phenomenon in proteins with different functions and structures, indicating that cotranslational assembly is widespread. Cotranslational assembly might be required for certain proteins to associate, or it might be important in cases where the early formation of a protein complex is beneficial, such as when a protein is toxic or unstable unless bound to a partner.

## Introduction

The majority of cellular proteins function as subunits in larger protein complexes. However, very little is known about how protein complexes form *in vivo*. One possibility is that proteins are fully translated and released into the cytoplasm before finding their interacting partners (posttranslational assembly). Alternatively, protein-protein interactions could form as one or several of the interacting proteins are being translated (cotranslational assembly).

There are indications that some cytoskeletal proteins, including vimentin, myosin and titin, assemble cotranslationally into insoluble filaments [1]. The formation of some multimeric membrane channels also appears to take place cotranslationally [2], [3]. There are also a few examples of cotranslational assembly of soluble proteins: the p53 and NF-κB transcription factors form homodimers, which are thought to be generated by cotranslational interactions within a single polysome [4], [5]. Importantly, the majority of these examples involve the assembly of a single protein into higher order structures. A number of recent studies have shown that the use of immunoprecipitation coupled with microarray analysis (RIp-chip, for Ribonucleoprotein Immunoprecipitation analysed with DNA chips) can be used to study cotranslational pathways involved in protein biosynthesis [6], [7], [8]. In this approach, a protein is purified together with associated RNAs, and the mRNAs are identified using DNA microarrays. When this method is applied to proteins associated with polysomes, it allows the identification of mRNAs cotranslationally associated with the bait protein. Using this technique we recently showed that the Rng3p myosin-specific chaperone associates cotranslationally with all five myosin heavy chains in the fission yeast *Schizosaccharomyces pombe* [6]. Another study in the budding yeast *Saccharomyces cerevisiae* found that the *SET1* mRNA is part of a complex containing four components of the SET1C histone methyltransferase complex. The protein-RNA interactions were dependent on active translation, suggesting that the complex between these proteins was formed cotranslationally [7]. Apart from these few examples, very little is known about the prevalence of cotranslational assembly in the formation of protein complexes. Importantly, systematic approaches to identify and characterise this phenomenon (such as RIp-chip) have not been applied to large numbers of proteins.

To address these questions we carried out RIp-chip experiments with 31 proteins with different functions and structures. We found that more than 12 of the proteins interacted specifically with small numbers of mRNAs (between 1 and 3), most of which encoded proteins that are known or predicted to interact with the bait proteins. We examined the protein-RNA interactions of three proteins in detail: in all cases we found that the interactions required the presence of the protein encoded by the associated mRNA as well as active translation. These data demonstrate that these protein mRNA interactions reflect the cotranslational formation of protein-protein interactions, and suggest that this is a widespread phenomenon.

## Results

### The Tea2p Kinesin and the Tip1 CLIP-170 protein interact cotranslationally

**Figure 1:**
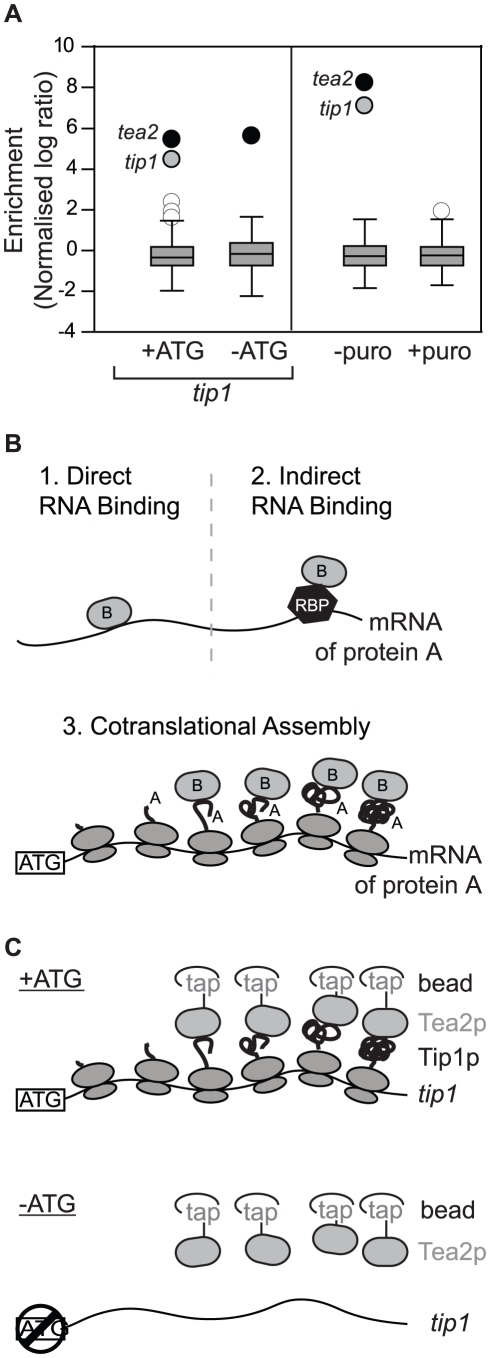
Cotranslational assembly of the Tea2p-Tip1p complex. (A) RIp-chip experiments with Tea2p. The y axis shows the log_10_ enrichment ratios in Tea2p RIp-chip experiments, standardised to make the mean and standard deviation equal to 0 and 1, respectively. The box plots show the distribution of enrichments, with the box showing the lower and upper quartiles, the whiskers representing data within the upper/lower quartile plus/minus 1.5-fold the interquartile range, and other data points displayed as circles. White circles represent mRNAs not considered significant (either because they are common contaminants in multiple RIp-chip experiments, or because they were not reproducibly enriched in independent replicas of the experiment), black circles correspond to mRNAs encoding the bait, and grey circles are used for mRNAs specifically associated with the bait protein. Left: Tea2p copurifies with wild type *tip1* (+ATG), but not with *tip1* that cannot be translated (−ATG). Right: The interaction between Tea2p and the *tip1* and *tea2* mRNAs is lost upon treatment of the cells with puromycin. (B) Three models to explain the association between Tea2p and the *tip1* mRNA (see text for details). (C) Design of the −ATG experiment coupled to RIp-chip analysis. A single nucleotide mutation to the start codon prevents the translation of *tip1*. If the association between Tea2p and *tip1* is cotranslational, lack of Tip1p should abolish their interaction.

As part of a project to identify RNAs associated with molecular motors in *S. pombe*, we performed RIp-chip experiments with the Tea2p kinesin. Surprisingly, Tea2p copurified specifically with only two mRNAs: *tea2* and *tip1* (Figure 1A). *tip1* encodes a protein of the CLIP-170 family that interacts physically with Tea2p and is transported by it along cytoplasmic microtubules [9]. We considered three models that could explain the association between Tea2p and the *tip1* mRNA: in models 1 and 2 (Figure 1B), Tea2p could interact with specific sequences on the *tip1* mRNA, either directly (model 1) or through a sequence-specific RNA-binding protein (model 2). In model 3, Tea2p interacts with the Tip1p nascent peptide, and thus pull downs the *tip1* mRNA as part of the whole polysome. In this case, the complex between Tea2p and Tip1p forms cotranslationally. The different models can be distinguished from each other experimentally by their dependence on the Tip1 protein for the interaction: in models 1 and 2 (recognition of RNA sequences), the interaction between Tea2p and *tip1* mRNA should be independent of the presence of Tip1p. In model 3 (cotranslational assembly), the association should only occur if Tip1p is present.

To discriminate between these possibilities we used a strain expressing *tip1* RNA but not Tip1 protein (Figure 1C, Figure S1, Figure S2). The strain was generated by mutating a single nucleotide in the initiation codon of *tip1* (*−ATG-tip1*), and expressing the resulting construct in cells in which the endogenous *tip1* gene had been deleted (see Materials and Methods). As a control, a similar strain was constructed expressing wild type *tip1* (+*ATG-tip1*). While Tea2p and *tip1* mRNA copurified in +*ATG-tip1* cells, the interaction was completely lost in *−ATG-tip1* cells (Figure 1A and Table S1). By contrast, *tea2* mRNA was precipitated to a similar extent in +*ATG-tip1* and *−ATG-tip1* cells (Figure 1A and Table S1). These data demonstrate that association of Tea2p with the *tip1* mRNA is dependent on expression of Tip1p, and strongly suggests that Tea2p and Tip1p bind to each other cotranslationally. This model makes a further prediction, namely that the interaction between Tea2p and *tip1* should be dependent on the integrity of polysomes. To test this idea we performed RIp-chip experiments after disrupting polysomes *in vivo* and *in vitro*. Treatment of *S. pombe* cells with puromycin leads to polysome disassembly and the release of nascent peptides [10], [11]. In cells incubated with puromycin, the interactions between Tea2p and both *tea2* and *tip1* mRNAs were entirely lost (Figure 1A and Table S1). Treatment of extracts with EDTA, which chelates magnesium and causes polysome disassembly, also disrupted the association between Tea2p and *tip1* and *tea2* mRNAs (Table S1). All together, these experiments strongly suggest that the complex between the Tea2 and Tip1 proteins forms cotranslationally. To test if this phenomenon is bidirectional (i.e. if Tip1p interacts with Tea2p as *tea2* mRNA is being translated), we carried out RIp-chip experiments with Tip1p. In this case, Tip1p coprecipitated with its own mRNA, but not with that of *tea2* (Figure S3 and Table S2).

### Many proteins associate with mRNAs encoding interacting proteins

**Figure 2:**
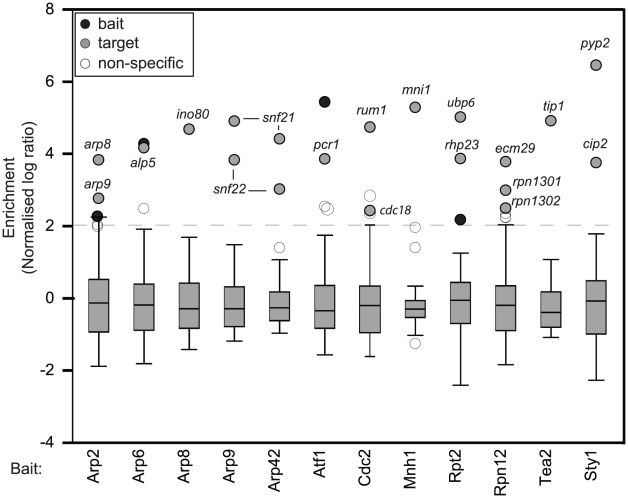
Many proteins that lack RNA–binding domains associate specifically with small numbers of mRNAs. Box plots of the distribution of enrichments for 12 RIp-chip experiments. The y axis shows normalised log_10_ enrichment ratios in the corresponding RIp-chip experiments (see Figure 1 for details). For each protein, a representative experiment is presented. Black circles show the mRNA encoded by the bait protein used for the RIp-chip experiment, and grey circles other mRNAs that were consistently enriched in independent biological experiments. White circles represent mRNAs not considered significant, either because they are common contaminants in multiple RIp-chip experiments, or because they were not reproducibly enriched in independent replicas of the experiment. The dashed line at two standard deviations above the mean shows the threshold used to define mRNA enrichment.

We wondered whether cotranslational assembly is a common mechanism for the formation of protein complexes. To address this question we analyzed 31 proteins by RIp-chip (Figure S3 and Table S2). We tested a variety of proteins, none of which contained canonical RNA-binding domains. Our baits included protein kinases, transcription factors, components of the proteasome, kinesins and several members of the actin related protein (Arp) family. Of the 31 bait proteins probed, 10 showed no significant association with any RNA, 9 proteins coprecipitated with only their own mRNAs, and 12 proteins reproducibly pulled down other mRNAs (Figure S3 and Table S2). Notably, the majority of associated mRNAs encoded known or suspected protein interactors of the corresponding bait proteins (Figure 2 and Table 1).

**Table 1:** Proteins analysed by RIp-chip and mRNAs associated with them.

Bait	Bait function/protein complex	Enriched mRNAs	Function of proteins encoded by interacting mRNAs
Tea2p	Kinesin motor protein [32]	*tip1*	CLIP170 family, binds to Tea2p [9]
Cdc2p	cyclin-dependent protein kinase [33]	*rum1* *cdc18*	CDK (cyclin-dependent kinase) inhibitor [12]DNA replication factor [13]
Sty1p(Spc1p)	MAP kinase; stress-responses [14], [34]	*pyp2* *cip2*	Tyrosine phosphatase, acts on Sty1p [14]RNA-binding protein [15]
Rpt2p (Mts2p)	19S proteasome regulatory subunit * [35]	*ubp6* *rhp23*	Ubiquitin C-terminal hydrolase * [35]Rad23 homolog * [35]
Rpn12p(Mts3p)	19S proteasome regulatory subunit * [35]	*ecm29* *rpn1301* *rpn1302*	Proteasome component * [35]19S proteasome regulatory subunit * [35]19S proteasome regulatory subunit * [35]
Atf1p	Transcription factor; stress response [36]	*pcr1*	Transcription factor, interacts with Atf1p [16]
Mnh1p	Mago nashi homolog; splicing * [35]	*mni1*	Protein with Mago nashi interacting domain * [35]
Arp6p	SWR1 complex; chromatin remodelling [18]	*alp5*	INO80 and SWR1 chromatin remodelling complexes [18]
Arp9p	SWI/SNF and RSC complex; chromatin remodelling [17]	*snf21* *snf22*	DNA helicase, RSC complex [17]DNA helicase, SWI/SNF complex [17]
Arp42p	SWI/SNF and RSC complex; chromatin remodelling [17]	*snf21* *snf22*	DNA helicase, RSC complex [17]DNA helicase, SWI/SNF complex [17]
Arp8p	Ino80 complex; chromatin remodelling [18]	*ino80*	INO80 chromatin remodelling complex [18]
Arp2p	Arp2/3 complex; actin polymerization [37]	*arp8* *arp9*	INO80 chromatin remodelling complex [18]SWI/SNF and RSC complexes [18]

Only proteins that copurified with mRNAs other than their own are shown. Proteins that have not been characterised in *S. pombe*, and for which the information is a prediction based on the behaviour of orthologous proteins are marked with a star.

A striking example is provided by Cdc2p, the ortholog of CDK1 in higher eukaryotes. Cdc2p is the only cyclin-dependent kinase in fission yeast and is an essential regulator of cell cycle progression. Cdc2p interacted with two mRNAs: *rum1*, which encodes a CDK (Cyclin-Dependent Kinase) inhibitor that associates with Cdc2p and inhibits its kinase activity [12], and *cdc18*, which encodes an essential DNA replication factor (a homologue of budding yeast *CDC6*) [13]. Both Cdc18p and Rum1p are also direct targets of Cdc2p.

Another protein kinase, Sty1p, which is a MAP kinase that mediates most stress responses in fission yeast, interacted with three mRNAs, *pyp2*, *cip2* and its own transcript. Pyp2p is a protein tyrosine phosphatase that directly binds and dephosphorylates Sty1p [14]. Cip2p is an RNA-binding protein thought to be regulated by Sty1p (however, no direct protein-protein interaction has been demonstrated) [15]. A predicted component of the 19S proteasome regulatory subunit, Rpn12p/Mts3p, associated with mRNAs encoding other subunits of the 19S proteasome (*rpn1301, rpn1302*) and a protein required for the assembly of the proteasome core and regulatory subunits (*ecm29*). A second component of the 19S proteasome, Rpt2p/Mts2p, interacted with the *ubp6* and *rhp23* mRNAs. Ubp6p is a proteasome-associated ubiquitin C-terminal hydrolase, while Rhp23p contains a ubiquitin-like N-terminus. The budding yeast orthologs of these two proteins (Ubp6 and Rad23, respectively) copurify with components of the proteasome. We also looked at two transcription factor of the b-ZIP family, Atf1p and Pcr1p, which can form heterodimers with each other [16]. Atf1p interacted with *pcr1* and its own mRNA, while Pcr1p only pulled down its cognate mRNA. Finally, Mnh1p, the *S. pombe* ortholog of the Mago nashi protein (*SPBC3B9.08c*, a component of the splicing-dependent exon–exon junction complex) associated with the mRNA of *mni1* (*SPBC19C7.01*), which encodes a protein that contains a Mago nashi-binding domain.

These data suggested that the mRNAs associated to a protein can provide insight into their cellular function and the protein complexes they belong to. To test this idea we examined all 10 members of the Actin-related protein family, which are structurally similar to actin but involved in functions as varied as chromatin remodelling, actin polymerisation or microtubular transport (as part of dynactin). These functions are carried out as part of different protein complexes that have been well characterised [17], [18], providing an excellent model to test this hypothesis. Half of the probed proteins pulled down specific mRNAs other than their own. The dynactin Arps (Arp1p and Arp10p) were the only members of this family that did not to associate with any mRNA. *S. pombe* nuclear Arps are components of several chromatin-remodelling complexes (SWI/SNF, INO80, NuA4 and Swr1C). Arp6p, a component of the Swr1C chromatin-remodelling complex, copurified with the *alp5* mRNA (Alp5p is also part of this complex, but also of Ino80 and NuA4) [18]. Arp9p and Arp42p are members of the SWI/SNF and RSC complexes [17], and both proteins associated with mRNAs encoding two SNF helicases (*snf21* and *snf22*). The Ino80 complex contains three Arps (Arp8p, Arp5p and Alp5p) [18]. Of these, Arp8p associated with the *ino80* mRNA, while Arp5p and Alp5p pulled down only their own mRNAs. Unexpectedly, Arp2p, a member of the family that regulates actin polymerisation, copurified with the mRNAs of two unrelated Arps (*arp8* and *arp9*). These proteins have very different functions and are not expected to interact directly with Arp2p. Therefore, the nature of the protein-RNA interactions of the Arps could allow the assignment of one protein (Arp6p) to one of several related complexes, and the unambiguous allocation of three proteins to a specific complex (Arp8p, Arp9p and Arp42p).

### Cotranslational formation of protein complexes is widespread

**Figure 3:**
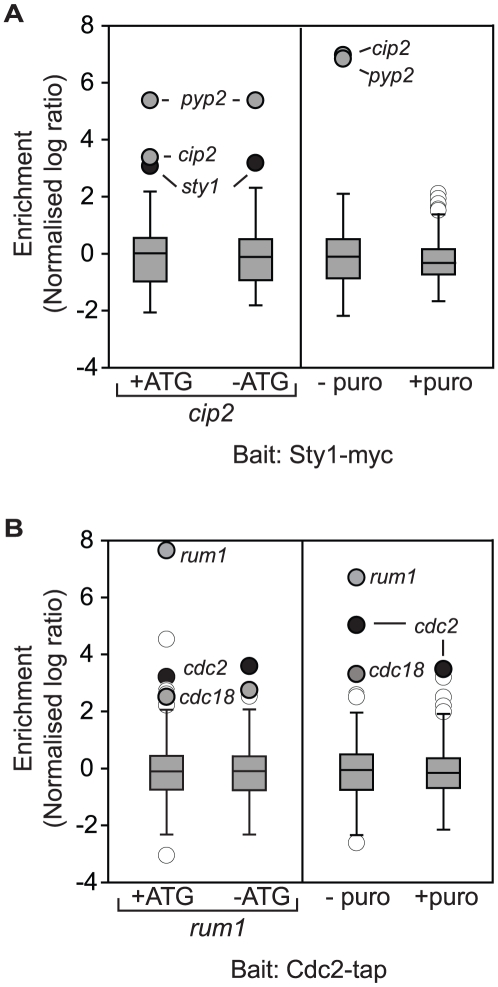
Cotranslational association of Cdc2p-Rum1p and Sty1p-Cip2p. Box plots of the distribution of normalised log_10_ enrichments for RIp-chip experiments with (A) Sty1p and (B) Cdc2p (see legend to Figure 1 for details). The left panels show the comparison between −ATG cells and the corresponding wild type controls. The right panels display the comparison between cells incubated with puromycin and a mock-treated control. mRNAs encoding the bait are shown in black, mRNAs not considered significant are displayed in white, and other significant mRNAs are presented in grey.

These results demonstrate that a large fraction of proteins copurify with mRNAs encoding interacting proteins. To confirm that, as in the case of Tea2p/*tip1*, these interactions reflect cotranslational formation of the corresponding protein complexes, we characterised in more detail the interactions between Sty1p/*cip2* and Cdc2p/*rum1*. We followed the same strategy described above for Tea2p/*tip1*. First, we made non-translatable versions of the *rum1* and *cip2* mRNAs (Figure S1 and Figure S2). Second, we performed RIp-chip experiments after *in vivo* treatment of the cells with puromycin, or after *in vitro* incubation of the extracts with EDTA. Sty1p did not associate with −ATG-*cip2*, while the association with another mRNA (*pyp2*) was unaffected by the mutation. By contrast, neither *cip2* nor *pyp2* copurified with Sty1p after puromycin or EDTA treatments (Figure 3A and Table S1). Similarly, the association between Cdc2p and *rum1* was lost in *−ATG-rum1*, but the interaction with *cdc18* was not. As expected, both associations were disrupted by puromycin and EDTA incubations (Figure 3B and Table S1).

## Discussion

Our data show that around 38% of a randomly selected set of proteins that do not contain canonical RNA-binding domains specifically copurify with small number of mRNAs (between 1 and 3). Remarkably, the majority of these mRNAs encode proteins that are closely related to the proteins used as bait, either as known direct interactors or as members of the same multiprotein complex. In some cases (*rum1*, *pyp2*), the proteins are key regulators of the bait protein. This phenomenon is not limited to a specific type of protein (we have observed it with protein kinases, transcription factors, actin-related proteins, kinesins, etc), nor is it restricted to specific cellular processes (the proteins we tested function in microtubular transport, cell cycle control, proteolysis, stress responses, chromatin remodelling and splicing). In fact, the functional association between bait proteins and their associated mRNAs could conceivably be used to gain insight into the function of the bait protein (if the proteins encoded by the interacting mRNAs have known functions) or to identify interacting partners. Although previous work had hinted at the importance of cotranslationally assembly (especially for homomultimers), this is the first demonstration that cotranslational assembly is a widespread and ubiquitous process.

We notice that the protein-mRNA interactions we report are extremely specific. For example, Cdc2p interacts with and is regulated by many proteins, including at least four cyclins, protein phosphatases (Cdc25p), protein kinases (Wee1p) and kinase inhibitors (Rum1p). In addition, Cdc2p is thought to have dozen of targets. However, Cdc2p associates specifically with two mRNAs. This suggests that only a fraction of all protein-protein interactions are formed cotranslationally. Interestingly, both proteins encoded by the Cdc2p-bound mRNAs are phosphorylated by Cdc2p and degraded through the same mechanism [19], possibly pointing at a role of cotranslational assembly in the control of protein stability. The preference for certain nascent peptides to undergo cotranslational assembly could simply be a reflection of their levels in the cell (which, in turn, would depend on the abundance of their cognate mRNAs and their translation rates). However, Cdc2p associates with *rum1* but not with *cdc13* (which encodes a B type cyclin that binds to Cdc2p), despite the fact that *rum1* mRNA levels are almost 6-fold lower that those of *cdc13* and both mRNAs are associated with similar number of ribosomes (3.7 for *rum1* and 4.2 for *cdc13*) [20]. Therefore, the specificity of cotranslational assembly seems to be conferred by factors other than the abundance of the interacting nascent chains.

It has recently been shown that many proteins that lack RNA-binding domains can interact directly with specific mRNA sets [21], [22], [23]. We do not believe that our observations reflect direct binding: first, most of the proteins identified in those studies interacted with much larger numbers of mRNAs; second, in the few cases in which those interactions were analysed more thoroughly, they were shown to be resistant to EDTA, arguing against cotranslational assembly [23]. By contrast, the three examples we analysed in detail using a variety of approaches were consistent with cotranslational assembly. It is formally possible that our results reflect direct binding to RNA that is dependent on active translation through an unidentified mechanism, but we consider this possibility highly unlikely.

The cotranslational formation of protein complexes could be required for multiple reasons in the cell. It is possible that some interactions can only form before a given member of the complex has folded completely. Indeed, it is relatively frequent that recombinant proteins need to be coexpressed in order for them to form a complex. Second, some proteins are unstable in the absence of their partner. In this case, cotranslational assembly would stabilise the protein by reducing the time during which a protein is susceptible to degradation. This explanation has been proposed for the SET1C histone methyltransferase complex [7]. Consistently, Pcr1p is unstable in the absence Atf1p [24]. Finally, some proteins could be toxic when not part of a complex. Again, early formation of a complex would reduce this potential toxicity.

Unexpectedly, we have found that a large fraction of protein-protein interactions are likely to be formed cotranslationally, and we demonstrate that the RIp-chip strategy can provide a genome-wide view of this phenomenon. The protein-RNA networks we present here add another layer of complexity to the formation and regulation of protein complexes in eukaryotic cells.

## Materials and Methods

### Yeast methods and experimental design

Standard methods were used for fission yeast growth and manipulation [25]. Proteins were TAP-tagged using a one-step PCR method in haploid cells except for *alp5* and *arp10*, where ∼400 nucleotides of ORF and 3′UTR sequences were cloned into the pFA6a-2xTap-Kan vector [26], [27]. All construct were transformed into haploid cells with the exceptions of *arp1* (integrated in *pat1* diploids) and *alp5* and *arp10* (transformed into wild type diploids). Successful tagging was verified by western blot as described below. All epitope-tagged strains grew normally and displayed normal cell shape, showing that the tagged proteins were functional. A complete list of the strains used in this work is presented in Table S3. Integration of constructs at the *leu1* locus was performed as described [28].

### Construction of *−ATG* mutants

All constructs were tagged integrated into the *leu1* locus of a strain in which the endogenous copy of the mutated gene had been deleted (Table S3). The length of the flanking sequences required to include endogenous 5′ and 3′ UTR was estimated from high throughput sequencing data [29]. For every gene two constructs were made, one containing a mutated ATG and a control carrying the normal initiation codon. The presence of the mutations was confirmed by sequencing. In all three cases, the control construct complemented the phenotype of the corresponding deletion strain (Figure S2).

The *cip2* ORF and 989 bp downstream were amplified by PCR from genomic DNA using primers that added a SalI and an EcoRI site to the 5′ and 3′ end, respectively, and cloned into pBluescript II. Two constructs were made: in the wild type control the 5′ primer contained the endogenous ATG, while in the −ATG one the ATG was mutated to AGG. The *cip2* promoter (560 bp) region was amplified by PCR as a KpnI/SalI fragment and cloned upstream of the ORF. The *leu1* gene was amplified by PCR as a XmaI/NotI and cloned into the vectors above. The plasmids were linearised with NruI before transformation into *leu1-32 cip2Δ* cells.

The *tip1* ORF and 154 bp downstream were amplified by PCR from genomic DNA as PacI/AscI fragment and cloned into pFA6a. Two constructs were made: in the wild type control the 5′ primer contained the endogenous ATG, while in the −ATG one the ATG was mutated to TTT. The *tip1* promoter region (865 bp) was amplified by PCR as a BamHI/PacI fragment and cloned into the vector above. The whole construct containing the *tip1* ORF and flanking regions was cloned into pJK148 (containing a *leu1* marker) as a KpnI/BamHI fragment. The construct was linearised and transformed as described above into *leu1-32 tip1Δ* cells.

For *rum1* we used site-directed mutagenesis to mutate the first five ATGs of the ORF to CTGs. However, we found that this construct was still able to complement the sterility phenotype of a *rum1Δ* strain. This suggested that translation was taking place from a cryptic start site (different from ATG). Therefore, we made a construct in which all seven in-frame ATGs in the *rum1* ORF were mutated to stop codons. For this construct a DNA fragment was synthesised (GENEART) that contained the whole ORF and flanking sequences (from the NheI to the SphI restriction sites), and in which all 7 in-frame ATGs had been replaced with the stop codon TGA. The digested DNA fragment was used to replace the corresponding section in pJET2.1 containing a *leu1* marker and 2.6 kb of *rum1* ORF and UTR sequences. −ATG and +ATG *rum1* plasmids were linearised with NruI and transformed into *leu1-32 rum1Δ* cells.

In contrast to the situation with *rum1*, mutation of the first ATG of *cip2* and *tip1* was sufficient to create a loss-of-function phenotype indistinguishable to that of the deletion mutant. As both genes contain several ATGs downstream of the annotated initiation codon, we cannot completely rule out the possibility that in the –ATG mutants there is initiation from downstream ATGs (or from other cryptic sites different from ATG), leading to the formation of N-terminally truncated peptides. However, any such peptides would not be functional (Table S3).

### Protein detection

Expression of TAP-tagged proteins was verified by western blot using peroxidase-anti-peroxidase soluble complexes (Sigma) to detect the protein A-binding domains of TAP. Myc tags were detected using the 9E11 monoclonal (Abcam).

### RIp-chip

We followed a previously published protocol [6]. Immunoprecipitation of TAP-tagged proteins was carried out using monoclonal antibodies against protein A (Sigma), and myc-tagged proteins were purified using the 9E11 monoclonal antibody (Abcam). All experiments were performed with vegetative cells except those using Arp1-TAP, Alp5-TAP and Arp10-TAP, which were carried out under meiotic conditions. Puromycin experiments were conducted by adding a final concentration of 1 mM puromycin to cell cultures, followed by incubation at 32°C for 15 minutes. In addition, immunoprecipitation buffers contained 1 mM puromycin and 2 mM GTP. In control experiments the buffers contained only 2 mM GTP. EDTA treatment was performed as previously described [6].

### Labelling and microarray experiments

Total RNA purified from the cell extract was used as a reference in all experiments. 20 µg of total RNA and all the RNA from the IP were labelled using the SuperScript Plus Direct cDNA Labelling System (Invitrogen). Labelled cDNAs were hybridised to PCR *S. pombe* DNA microarrays or to custom-designed oligonucleotide microarrays manufactured by Agilent as described [30], [31]. Microarrays were scanned with a GenePix 4000A microarray scanner and analysed with GenePix Pro 5.0 (Molecular Devices).

### Data analysis

Only probes corresponding to coding sequences were considered. Spots with unreliable signals were removed as follows: for the PCR microarrays, spots that did not show a minimum of 55% of pixels above the median background signal plus two standard deviations in the immunoprecipitate and 90% of pixels in the total RNA (or at least 90% in the channel of the IP) were removed; for the Agilent microarrays the corresponding thresholds were 70%, 98% and 98%, respectively. Median log_10_ ratios were used for the analysis. We compiled a list of common mRNA unspecific contaminants, which were removed from the analysis. Selection of enriched RNAs in the RIp-chip experiments was carried out as described [6], by choosing genes whose enrichment ratios were at least two standard deviations above the median enrichment of all genes. Only mRNAs that passed this threshold in every independent biological experiment were considered enriched (Table 1). Assuming a normal distribution of the enrichments, the expected fraction of false positives using this threshold with a single experiment would be ∼0.05. However, as we only selected RNAs enriched in each of 2–4 repeats, this number is reduced to ∼2.5×10^−3^ to ∼6.25×10^−6^. All RIp-chip experiments in which mRNAs different from the one encoding the bait were detected were carried out at least twice. Other RIp-chips (negatives or containing only the cognate mRNA of the bait) were performed once or twice. All repeats were independent biological experiments and dyes were swapped for at least one experiment with each protein.

### Data deposition

All raw and normalised microarray data have been deposited in ArrayExpress (accession number E-TABM-1158). Dataset S1 contains normalised data and experimental details for all RIp-chip experiments reported in this manuscript.

## Supporting Information

Figure S1 Non-translatable mRNAs are expressed at levels comparables to wild-type mRNAs. Raw microarray signals of total wild type mRNAs (+ATG) compared to untranslatable mRNAs (−ATG). The data are shown for every probe of the microarray and have not been normalised or filtered to remove weak signals. The number of independent probes varies between 2 and 5 depending on the microarray platform used for the experiment. We detected strong signals for all the −ATG constructs, ruling out the possibility that the mRNAs encoding untranslatable proteins are degraded.
(PDF)

Figure S2 No functional proteins are produced from the untranslatable mRNAs. (A) Spot assays to measure sensitivity to oxidative stress. Fourfold serial dilutions were plated on yeast extract (YES) plates (Control) or YES plates containing 1 mM H_2_O_2_. *csx1*Δ cells are sensitive to oxidative stress. This phenotype is suppressed by deletion of c*ip2*. The presence of the *−ATG-cip2* construct did not affect the phenotype of *cip2Δ*, indicating that no functional Cip2 protein was produced. (B) Morphology of *tip1*Δ mutants. *tip1Δ* cells containing the *−ATG-tip1* construct or the corresponding wild type control (+ATG) were grown for 48 hours in YES and inoculated into fresh medium. *−ATG-tip1* cells showed the characteristic branched morphology of *tip1* null mutants, while the wild type control suppressed the *tip1Δ* phenotype. Scale bars: 10 µm. (C) Sterility of *rum1Δ* mutants. *rum1Δ* expressing *−ATG-rum1* or the corresponding wild type control (+ATG) were incubated on malt extract plates for 48 hours. *−ATG-rum1* did not suppress the sterility phenotype of *rum1Δ* cells, whereas *rum1Δ* cells with the +ATG construct mated and sporulated normally. Scale bars: 10 µm.
(PDF)

Figure S3 RIp-chip experiments with 31 proteins. The box plots show the distribution of the enrichments for each of the experiments reported in this paper. The y axis shows normalised log_10_ enrichment ratios in the corresponding RIp-chip experiments (see legend to Figure 1 for details). For each protein, a representative experiment is presented. Black circles show the mRNA encoding the bait protein used for the RIp-chip experiment, and grey circles other mRNAs that were consistently enriched in independent biological experiments. White circles represent mRNAs not considered significant, either because they are common contaminants in multiple RIp-chip experiments, or because they were not reproducibly enriched in independent replicas of the experiment. The dashed line at two standard deviations shows the threshold we used to determine significant enrichment. The results are shown for proteins that did not copurify specifically with any mRNAs (‘empty’), those that associated only with their cognate mRNA (‘bait only’) and those that bound to other mRNAs (‘targets’).
(PDF)

Table S1 mRNA enrichments in puromycin, EDTA and ΔATG experiments. The numbers display the number of standard deviations above the median enrichment of all mRNAs in the immunoprecipitate (see Methods). Multiple numbers correspond to independent biological replicates of the experiment. Cases where there were not enough mRNAs in the IP to calculate the median enrichment for the background distribution, but in which the corresponding mRNA was clearly present, are denoted as P. mRNAs that were not detectable in the immunoprecipitate are indicated as ND. The mRNAs used for the ΔATG experiments are underlined.
(PDF)

Table S2 mRNA enrichments in all RIp-chip experiments. Only mRNAs considered significant are displayed. The numbers represent the number of standard deviations above the median enrichment of all mRNAs in the immunoprecipitate (see Methods). For experiments performed three times or less the enrichment in each biological replicate is indicated. For other experiments the numbers indicate the average and standard deviation of the enrichments.
(PDF)

Table S3 Strains used in this study. YGRC: Yeast Genetic Resource Center, Osaka City University (Japan).
(PDF)

Dataset S1 Normalised enrichment levels for all RIp-chip experiments.
(XLS)
