# Supplementary figures and images for: Widespread Cotranslational Formation of Protein Complexes
Source: PLoS Genet. 2011 Dec 1;7(12):e1002398. doi: 10.1371/journal.pgen.1002398 (PMC3228823; doi:10.1371/journal.pgen.1002398)

(A)

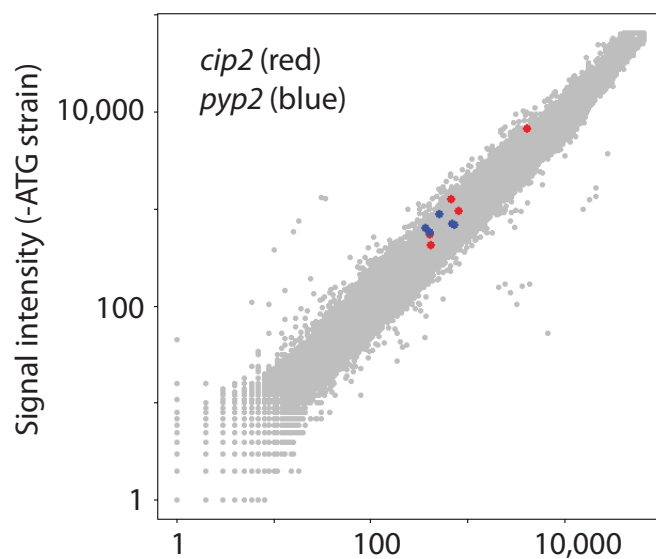

(B)

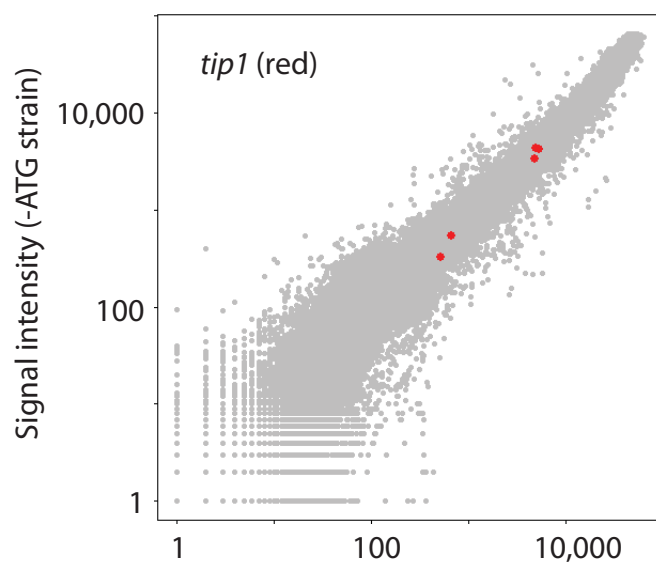

(C)

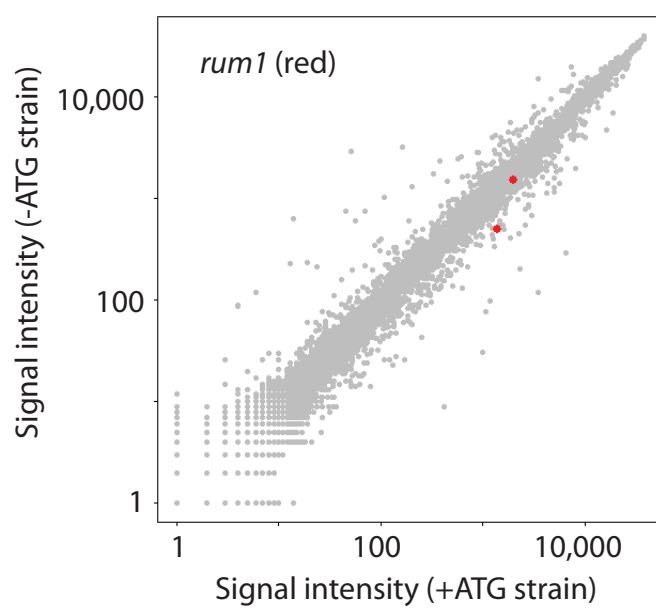

Supplement: Figure S1 — Non-translatable mRNAs are expressed at levels comparables to wild-type mRNAs. Raw microarray signals of total wild type mRNAs (+ATG) compared to untranslatable mRNAs (−ATG). The data are shown for every probe of the microarray and have not been normalised or filtered to remove weak signals. The number of independent probes varies between 2 and 5 depending on the microarray platform used for the experiment. We detected strong signals for all the −ATG constructs, ruling out the possibility that the mRNAs encoding untranslatable proteins are degraded. (PDF) [file pgen.1002398.s001.pdf]

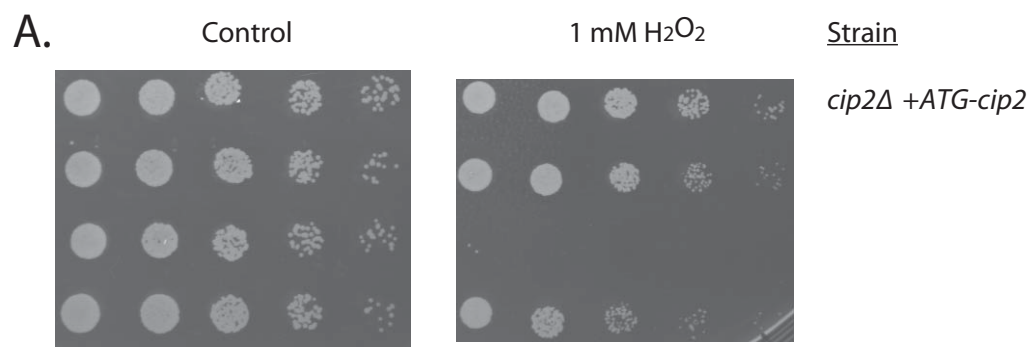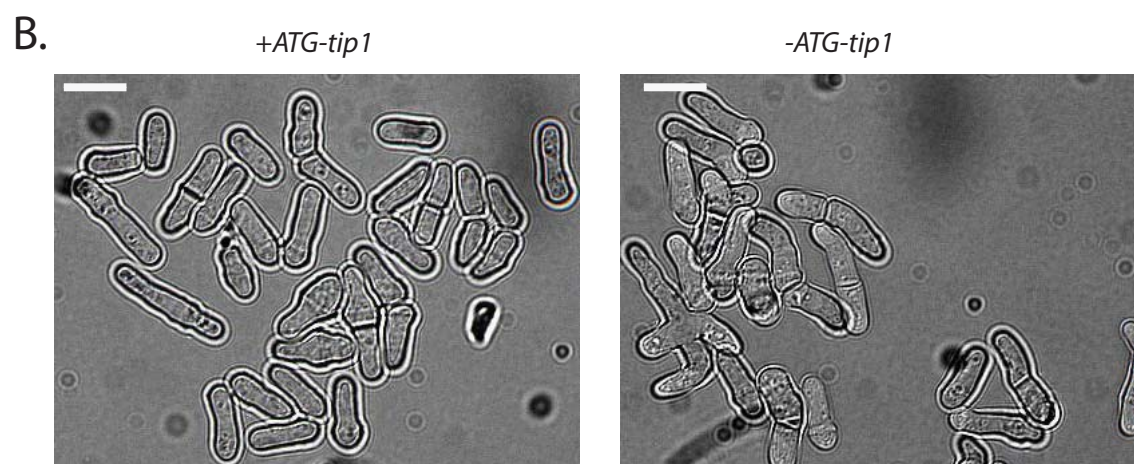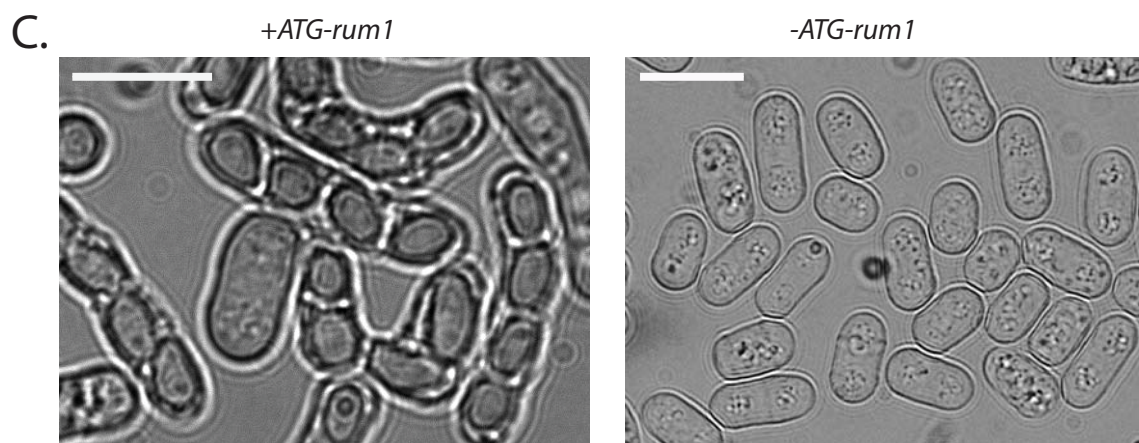

Supplement: Figure S2 — No functional proteins are produced from the untranslatable mRNAs. (A) Spot assays to measure sensitivity to oxidative stress. Fourfold serial dilutions were plated on yeast extract (YES) plates (Control) or YES plates containing 1 mM H2O2. csx1Δ cells are sensitive to oxidative stress. This phenotype is suppressed by deletion of cip2. The presence of the −ATG-cip2 construct did not affect the phenotype of cip2Δ, indicating that no functional Cip2 protein was produced. (B) Morphology of tip1Δ mutants. tip1Δ cells containing the −ATG-tip1 construct or the corresponding wild type control (+ATG) were grown for 48 hours in YES and inoculated into fresh medium. −ATG-tip1 cells showed the characteristic branched morphology of tip1 null mutants, while the wild type control suppressed the tip1Δ phenotype. Scale bars: 10 µm. (C) Sterility of rum1Δ mutants. rum1Δ expressing −ATG-rum1 or the corresponding wild type control (+ATG) were incubated on malt extract plates for 48 hours. −ATG-rum1 did not suppress the sterility phenotype of rum1Δ cells, whereas rum1Δ cells with the +ATG construct mated and sporulated normally. Scale bars: 10 µm. (PDF) [file pgen.1002398.s002.pdf]

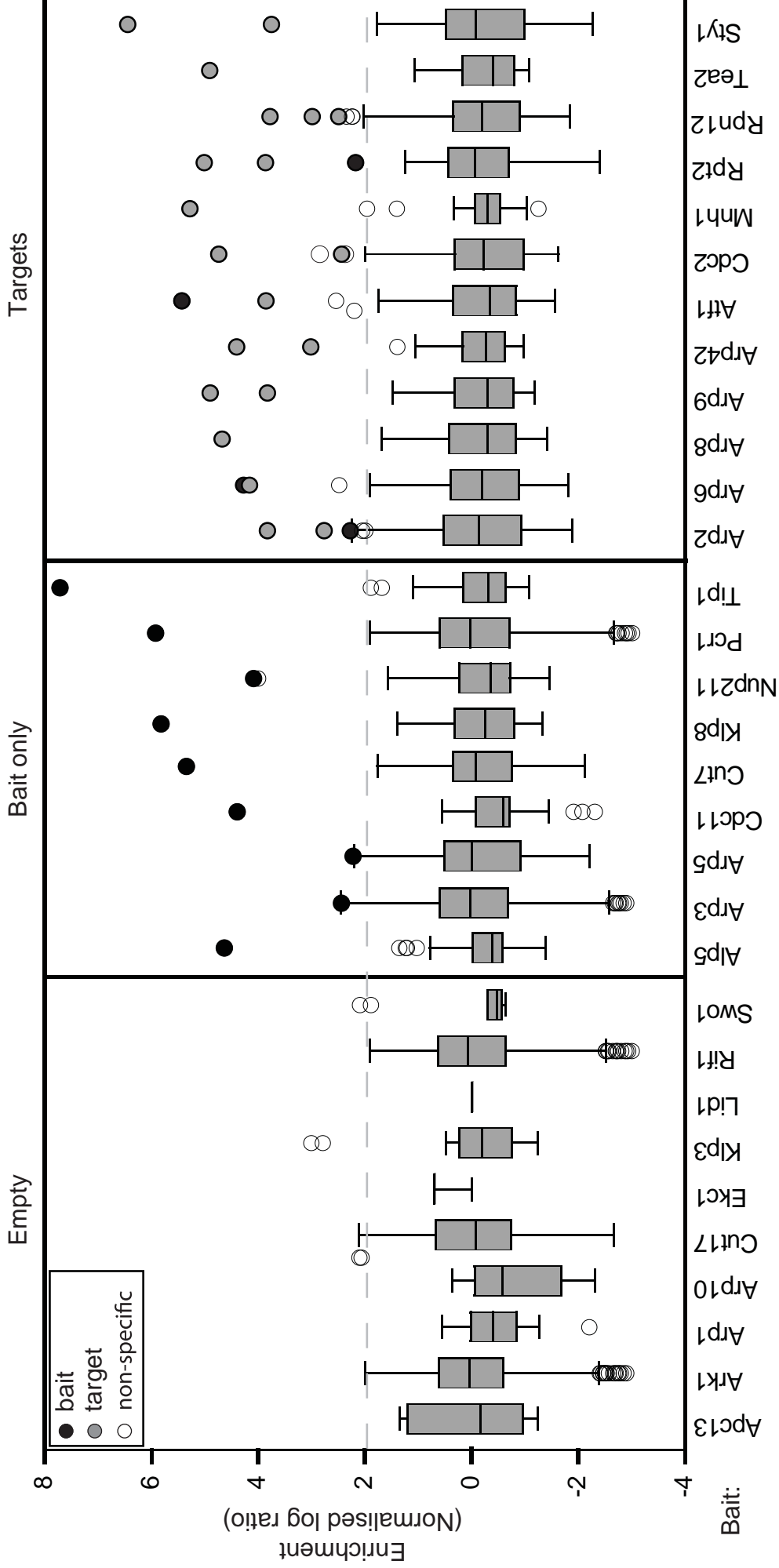

Supplement: Figure S3 — RIp-chip experiments with 31 proteins. The box plots show the distribution of the enrichments for each of the experiments reported in this paper. The y axis shows normalised log10 enrichment ratios in the corresponding RIp-chip experiments (see legend to Figure 1 for details). For each protein, a representative experiment is presented. Black circles show the mRNA encoding the bait protein used for the RIp-chip experiment, and grey circles other mRNAs that were consistently enriched in independent biological experiments. White circles represent mRNAs not considered significant, either because they are common contaminants in multiple RIp-chip experiments, or because they were not reproducibly enriched in independent replicas of the experiment. The dashed line at two standard deviations shows the threshold we used to determine significant enrichment. The results are shown for proteins that did not copurify specifically with any mRNAs (‘empty’), those that associated only with their cognate mRNA (‘bait only’) and those that bound to other mRNAs (‘targets’). (PDF) [file pgen.1002398.s003.pdf]
