# Supplementary material for: Widespread Cotranslational Formation of Protein Complexes
Source: PLoS Genet. 2011 Dec 1;7(12):e1002398. doi: 10.1371/journal.pgen.1002398 (PMC3228823; doi:10.1371/journal.pgen.1002398)
Supplement: Table S1 — mRNA enrichments in puromycin, EDTA and ΔATG experiments. The numbers display the number of standard deviations above the median enrichment of all mRNAs in the immunoprecipitate (see Methods). Multiple numbers correspond to independent biological replicates of the experiment. Cases where there were not enough mRNAs in the IP to calculate the median enrichment for the background distribution, but in which the corresponding mRNA was clearly present, are denoted as P. mRNAs that were not detectable in the immunoprecipitate are indicated as ND. The mRNAs used for the ΔATG experiments are underlined. (PDF) [file pgen.1002398.s004.pdf]

**Bait Protein**	**mRNA Target**	**-Puro**	**+Puro**	**-EDTA**	**+EDTA**	**+Target Protein**	**-Target Protein**
Tea2-TAP	*tip1*	7.1	ND	5.0, P	ND, ND	6.0, 4.4	ND, ND
	tea2	8.2	ND	5.4, P	ND, ND	5.5, 6.1	5.6, 5.9
Sty1-myc	*cip2*	7.0	ND	6.3, 3.8	ND, ND	2.1, 3.1	1.5, ND
	pyp2	6.8	ND	ND, 6.4	ND, ND	4.8, 5.4	4.9, 5.4
Cdc2-TAP	*rum1*	6.7	ND	8.8, 5.5	0.31, ND	7.6,6.4	0.36,0.51
	cdc18	3.2	ND	ND, 3.4	ND, ND	2.5, 3.3	2.7, 3.6
