# Supplementary material for: Widespread Cotranslational Formation of Protein Complexes
Source: PLoS Genet. 2011 Dec 1;7(12):e1002398. doi: 10.1371/journal.pgen.1002398 (PMC3228823; doi:10.1371/journal.pgen.1002398)
Supplement: Table S2 — mRNA enrichments in all RIp-chip experiments. Only mRNAs considered significant are displayed. The numbers represent the number of standard deviations above the median enrichment of all mRNAs in the immunoprecipitate (see Methods). For experiments performed three times or less the enrichment in each biological replicate is indicated. For other experiments the numbers indicate the average and standard deviation of the enrichments. (PDF) [file pgen.1002398.s005.pdf]

Bait Protein	Enriched mRNAs (SDs)	Function of the bait protein
Apc13-TAP		Anaphase-promoting complex subunit
Ark1-myc		Aurora-B kinase
Arp10-TAP		Dynactin subunit
Arp1-TAP		Dynactin subunit
Arp2-TAP	*arp8* (3.8, 4.1, 2.9)<br>*arp9* (2.8, 2.9, 2.4)	Member of Arp2/3 complex; involved in actin polymerization
Arp3-TAP	*arp3* (3.3, 2.5)	Member of Arp2/3 complex; involved in actin polymerization
Arp42-TAP	*snf21* (4.4, 4.7)<br>*snf22* (3.0, 3.6)	SWI/SNF and RSC complexes; chromatin remodelling
Alp5-TAP	*alp5* (4.6, 6.6)	NuA4, Ino80 and Swr1 complexes; chromatin remodelling
Arp5-TAP	*arp5* (5.8, 2.2)	Ino80 complex; chromatin remodelling
Arp6-TAP	*alp5* (4.2, 4.2, 3.8)<br>*arp6* (4.3, 2.4, 3.8)	SWR1 complex; chromatin remodelling
Arp8-TAP	*ino80* (4.7, 2.3)	Ino80 complex; chromatin remodelling
Arp9-TAP	*snf21* (4.8, 4.9)<br>*snf22* (2.7, 3.8)	SWI/SNF and RSC complexes; chromatin remodelling
Atf1-myc	*atf1* (5.4, 4.3, 4.5)<br>*pcr1* (3.9, 2.7, 2.0)	Transcription factor; stress responses
Cdc11-TAP	*cdc11* (4.4)	SIN complex scaffold protein
Cdc2-TAP	*rum1* (5.8±2.1, n=7)<br>*cdc18* (3.1±0.5, n=6)	Cyclin-dependent protein kinase
Cut17-myc		Survivin protein
Cut7-TAP	*cut7* (5.3)	Spindle pole body motor
Ekc1-myc		Protein phosphatase regulatory subunit
Klp3-TAP		Kinesin-like protein
Klp8-TAP	*klp8* (5.8)	Kinesin-like protein
Lid1-TAP		Anaphase-promoting complex subunit
Rpt2-myc	*ubp6* (5.0, 4.9)<br>*rhp23* (3.9, 3.7)	19S proteasome regulatory subunit
Rpn12-myc	*ecm29* (3.8, 3.6)<br>*rpn1301* (2.2, 2.9)<br>*rpn1302* (3.0, 2.6)	19S proteasome regulatory subunit
Nup211-TAP	*nup211* (4.1, 11.2)	Nucleoporin
Pcr1-myc	*pcr1* (5.9, 4.8, 4.1)	Transcription factor; stress responses
Rif1-myc		Telomere length regulator
Mnh1-TAP	*SPBC19C7.01/mni1*<br>(4.4, 5.3)	Mago nashi homolog
Sty1-myc	*pyp2* (5.4±0.8, n=8)<br>*cip2* (4.9±1.4, n=6)	MAP kinase; stress responses
Swo1-TAP		Hsp90 chaperone
Tea2-TAP	*tip1* (5.2±1.5, n=7)	Kinesin-like protein
Tip1-TAP	*tip1* (7.7)	CLIP-170 family proteins
