# Supplementary material for: Widespread Cotranslational Formation of Protein Complexes
Source: PLoS Genet. 2011 Dec 1;7(12):e1002398. doi: 10.1371/journal.pgen.1002398 (PMC3228823; doi:10.1371/journal.pgen.1002398)
Supplement: Table S3 — Strains used in this study. YGRC: Yeast Genetic Resource Center, Osaka City University (Japan). (PDF) [file pgen.1002398.s006.pdf]

Genotype	Source
apc13-TAP::kanMX6 ade6-M210 leu1-32 ura4-D18 h-	K. Gould (Vanderbilt University)
alp5-2xTAP::kanMX6/+ ade6-M210/ade6-M216 h+/h+	This work
ark1-8xmyc-6xhis:LEU2 leu1-32 h-	YGRC
arp1-2xTAP::kanMX6/+ pat1-114/pat1-114 ade6-M210/ade6-M216 h+/h+	This work
arp2- 2xTAP::kanMX6 h+	This work
arp3-2xTAP::kanMX6 h+	This work
arp42-2xTAP::kanMX6 h+	This work
arp5-2xTAP::kanMX6 h+	This work
arp6-2xTAP::kanMX6/+ ade6-M210/ade6-M216 h+/h+	This work
arp8-2xTAP::kanMX6 h-	This work
arp9-2xTAP::kanMX6 h-	This work
arp10-2xTAP::kanMX6/+ h+/h-	This work
atf1-13xmyc::kanMx6 leu1-32 ura4-D18 his7 ade6-216 h-	N. Jones (Manchester, UK)
cdc11-TAP::kanMX6 ade6-M210 ura4-D18 leu1-32 h-	K. Gould (Vanderbilt University)
cdc2-TAP::kanMX6 ade6-M210 ura4-D18 leu1-32 h-	K. Gould (Vanderbilt University)
cdc2-TAP::kanMX6 rum1Δ::ura4+ ura4-D18 leu1-32 ade6-M210 h90	This work
cdc2-TAP::kanMX6 rum1Δ::ura4+ pJK148-rum1-ATG+::leu1+ ura4-D18 leu1-32 ade6-M210 h90	This work
cdc2-TAP::kanMX6 rum1Δ::ura4+ pJK148-rum1-ATG-5xΔ::leu1+ ura4-D18 leu1-32 ade6-M210 h90	This work
cip2Δ::natMX6 leu1-32 ura4-D18 ade6-M21? h+	This work
cut7-2xTAP::kanMX6 h+	This work
cut17-8xmyc::LEU2 leu1-32 h-	YGRC
ekc1-myc::ura4+ leu1-32 ura4-D18 h-	YGRC
klp3-2xTAP::kanMX6 ade6-M216	This work
klp8-2xTAP::kanMX6 ade6-M216	This work
lid1-TAP ade6-M210 leu1-32 ura4-D18 h-	K. Gould (Vanderbilt University)
mnh1-2xTAP:kanMX6 (SPBC3B9.08c)	This work
rpt2-8xmyc::LEU2 leu1-32 h-	YGRC
rpn12-8xmyc::LEU2 leu1-32 h-	YGRC
nup211-2xTAP::kanMX6 h90	This work

pcr1-12xmyc::kanMx6 h-	N. Jones (Manchester, UK)
rif1-12xmyc::ura4+ ade6-M210 leu1-32 ura4-D18 h90	YGRC
rum1::ura4+ ura4-D18 leu1-32 ade6-M210 h90	S. Moreno (Salamanca, Spain)
sty1-12xmyc::kanMX6 cip2Δ::natMX6 leu1-32 ade6-M21? pJK148-cip2-ATG+::leu1+ h+	This work
sty1-12xmyc::kanMX6 cip2Δ::natMX6 leu1-32 ade6-M21? pJK148-cip2-ATG+::leu1+ csx1Δ::hygMX6 leu1-32	This work
sty1-12xmyc::kanMX6 cip2Δ::natMX6 leu1-32 ade6-M21? pJK148-cip2-ATGΔ::leu1+ csx1Δ::hygMX6 leu1-32	This work
sty1-12xmyc::kanMX6 cip2Δ::natMX6 leu1-32 ade6-M21? pJK148-cip2-ATGΔ::leu1+ h+	This work
sty1-12xmyc::kanMX6 cip2Δ::natMX6 leu1-32 ade6-M21? h+	This work
sty1-12xmyc::kanMX6 leu1-32 ade6-M216 h-	YGRC
swo1- 2xTAP::kanMX6 h90	This work
tea2-2xTAP::hygMX6 tip1Δ::kanMX4 leu1-32 h-	This work
tea2-2xTAP::hygMX6 tip1Δ::kanMX4 leu1-32 pJK148-tip1-ATG+::leu1+ h-	This work
tea2-2xTAP::hygMX6 tip1Δ::kanMX4 leu1-32 pJK148-tip1-ATGΔ::leu1+ h-	This work
tea2-2xTAP::kanMX6 h90	This work
tip1Δ::kanMX4 leu1-32 ade6-M21? ura4-D18 h+	J. Hayles (London, UK)
tip1-2xTAP::kanMX6 ade6-M216 h-	This work
